# RETRACTION: Effect of Possible Risk Factors for Pharyngocutaneous Fistula after Total Laryngectomy of Laryngeal Carcinomas and Surgical Wound Infection: A Meta‐Analysis

**DOI:** 10.1111/iwj.70752

**Published:** 2025-08-26

## Abstract

**RETRACTION**: ChangX. and HuY. , “Effect of Possible Risk Factors for Pharyngocutaneous Fistula after Total Laryngectomy of Laryngeal Carcinomas and Surgical Wound Infection: A Meta‐Analysis,” International Wound Journal 20, no. 7 (2023): 2664–2672, 10.1111/iwj.14140.37243402 PMC10410319

The above article, published online on 26 May 2023 in Wiley Online Library (http://onlinelibrary.wiley.com/), has been retracted by agreement between the journal Editor in Chief, Professor Keith Harding; and John Wiley & Sons, Ltd. An investigation by the publisher found that the Methods section lacked appropriate detail and thus was not reproducible. In Section 2.1 Reference 5 was found to be irrelevant and did not support the statements regarding eligibility criteria. Additionally, the article states that they have performed a sensitivity analysis in Section 2.8, but the article does not include a sensitivity analysis. Additionally, the review found that the article contained significant textual overlap in the methods section between this article and other articles by different authors [1‐3]. The authors responded to an inquiry by the publisher, but they were not able to provide a response to these concerns. As such, the retraction has been agreed to because the methodology contains missing information and is not reproducible. The authors stated that they agree to the retraction voluntarily.

**References:**

[1] 
CaoX.
, 
GengX.
, 
ZhangC.
, 
ChenJ.
, 
ZhangC.
, 
LiuQ.
, 
WuT.
, and 
LiL.
, “Effect of Instant Surgery Compared with Traditional Management on Paediatric Complicated Acute Appendicitis Post‐Surgery Wound: A Meta‐Analysis,” International Wound Journal
20, no. 8 (2023): 2964–2972, 10.1111/iwj.14163.36965159
PMC10502279

[2] 
WangY.
, 
WangS.
, and 
YangX.
, “Prevalence of Different Types of Wound Infection in Subjects with Parkinson's Disease and Total Joint Arthroplasty: A Meta‐Analysis,” International Wound Journal
20, no. 7 (2023): 2780–2787, 10.1111/iwj.14154.36924416
PMC10410355

[3] 
HongJ.
, 
XieL.
, 
FanL.
, and 
HuangH.
, “The Wound Adjuncts Effect of Closed Incision Negative Pressure Wound Therapy on Stopping Groin Surgical Site Wound Infection in Arterial Surgery: A Meta‐Analysis,” International Wound Journal
20, no. 7 (2023): 2726–2734, 10.1111/iwj.14146.36977282
PMC10410315

**RETRACTION**: X. Chang and Y. Hu , “Effect of Possible Risk Factors for Pharyngocutaneous Fistula after Total Laryngectomy of Laryngeal Carcinomas and Surgical Wound Infection: A Meta‐Analysis,” International Wound Journal 20, no. 7 (2023): 2664–2672, 10.1111/iwj.14140.37243402 PMC10410319

The above article, published online on 26 May 2023 in Wiley Online Library (http://onlinelibrary.wiley.com/), has been retracted by agreement between the journal Editor in Chief, Professor Keith Harding; and John Wiley & Sons, Ltd. An investigation by the publisher found that the Methods section lacked appropriate detail and thus was not reproducible. In Section 2.1 Reference 5 was found to be irrelevant and did not support the statements regarding eligibility criteria. Additionally, the article states that they have performed a sensitivity analysis in Section 2.8, but the article does not include a sensitivity analysis. Additionally, the review found that the article contained significant textual overlap in the methods section between this article and other articles by different authors [1‐3]. The authors responded to an inquiry by the publisher, but they were not able to provide a response to these concerns. As such, the retraction has been agreed to because the methodology contains missing information and is not reproducible. The authors stated that they agree to the retraction voluntarily.
